# 3D Clumped Cell Segmentation Using Curvature Based Seeded Watershed

**DOI:** 10.3390/jimaging2040031

**Published:** 2016-11-05

**Authors:** Thomas Atta-Fosu, Weihong Guo, Dana Jeter, Claudia M. Mizutani, Nathan Stopczynski, Rui Sousa-Neves

**Affiliations:** 1 Department of Mathematics, Applied Mathematics and Statistics, Case Western Reserve University, 10900 Euclid Avenue, Cleveland, OH 44106, USA; 2 Department of Biology, Case Western Reserve University, 10900 Euclid Avenue, Cleveland, OH 44106, USA; 3 Department of Genetics and Genome Science, Case Western Reserve University, 10900 Euclid Avenue, Cleveland, OH 44106, USA

**Keywords:** watershed transform, watershed, manifold, Weingarten map, shape operator, Gaussian curvature, mean curvature, catchment basin, topographic distance

## Abstract

Image segmentation is an important process that separates objects from the background and also from each other. Applied to cells, the results can be used for cell counting which is very important in medical diagnosis and treatment, and biological research that is often used by scientists and medical practitioners. Segmenting 3D confocal microscopy images containing cells of different shapes and sizes is still challenging as the nuclei are closely packed. The watershed transform provides an efficient tool in segmenting such nuclei provided a reasonable set of markers can be found in the image. In the presence of low-contrast variation or excessive noise in the given image, the watershed transform leads to over-segmentation (a single object is overly split into multiple objects). The traditional watershed uses the local minima of the input image and will characteristically find multiple minima in one object unless they are specified (marker-controlled watershed). An alternative to using the local minima is by a supervised technique called seeded watershed, which supplies single seeds to replace the minima for the objects. Consequently, the accuracy of a seeded watershed algorithm relies on the accuracy of the predefined seeds. In this paper, we present a segmentation approach based on the geometric morphological properties of the ‘landscape’ using curvatures. The curvatures are computed as the eigenvalues of the Shape matrix, producing accurate seeds that also inherit the original shape of their respective cells. We compare with some popular approaches and show the advantage of the proposed method.

## 1. Introduction

Image segmentation is a very important aspect of image processing, and plays vital roles in scientific research. One such area is transcription of gene expression in a cell. Researchers in Biology and Chemistry are often interested in which genes are expressed in each cell of an organism, as well as the physical properties of cells, such as volume and density ratio of proteins within the cells. One major challenge arises from the fact that cells may appear in different shapes and sizes, and are often clumped together in colonies making it very difficult to segment an image data of cells. This is even more challenging for 3D image data, due to the different morphology of the cells, for example, cell boundaries across the third (z-) dimension. Other mechanical limitations such as low resolution (imaging at high resolution can be very costly in terms of data size) of the imaging device also make it more challenging as it results in poorly defined boundaries and contrast variation within cell bodies. Attempts to automate this process of cell characterization has attracted several techniques of cell segmentation, such as the the *K*−means [1], fuzzy threshold method [2], and level set methods [3,4]. Each of these techniques has a known drawback when segmenting touching cells. For instance, the fuzzy threshold method relies on intensity thresholds for segmentation and given the brightness similarities on or near the boundaries of touching cells, it is usually difficult to obtain cell boundaries with the generated threshold. The level set methods similarly aim to enclose cells with a given level set which is propagated by a given force towards such boundaries. This method underperforms when cells are touching, since the forces are not accurate near boundaries. Moreover, due to the varied morphology of 3D cells, for a reasonable segmentation of such images, a slice by slice segmentation fails since boundaries of individual cells generally do not align in a fixed (z-) direction. This is a major drawback to some of the popular toolkits such as CellProfiler and CellSegm, and one needs sophisticated characterization and modeling to circumvent challenges ranging from noise to cell proliferation. An equally challenging task arises when objects substantially change shape across successive frames. CellProfiler uses a slice by slice segmentation for 3D image data, with obvious limitations for clumped cell segmentation. The segmentation pipeline for Dapi-stained nuclei in the CellSegm toolbox uses adaptive thresholding to find seeds for watershed segmentation, and then followed by cell splitting (if desired) [5] and we compare the proposed method (which is cast into a toolbox) with the performance of CellSegm and two other methods, MINS and SMMF, in [6,7] respectively, on both simulated and real image data of 3D cells.

One of the most widely used technique for segmenting touching cells is the watershed transform, which partitions an image into catchment basins separated by watershed lines along the boundaries of the cells, where the interior of the cells corresponds to the catchment basins. The most widely used form of the watershed segmentation technique relies on a region growing approach whereby initial locations within cells, also known as seeds, are expanded until boundaries of adjacent cells are reached [7–10].

The Watershed transform, as a region-based segmentation method [11], offers an intuitive approach for segmenting closely packed objects in an image. In mathematical morphology, a gray-scale image may be interpreted as a geographic landscape, where the elevation is usually represented by the intensity. This landscape can be separated into adjacent flooded basins with watersheds lines dividing the basins [12]. (For H&E stained images with dark-colored nuclei, the basins naturally identify with nuclei, while for others like Dapi stained nuclei, where nuclei locations have bright intensities, the gradient of the image will match nuclei regions with the basin). Therefore the ability to correctly identify the basins is a very important step in accurately labeling the landscape into regions. A very popular step in this process is seed/marker detection, one for each nuclei, from which the corresponding catchment basin is grown. In the traditional watershed algorithm, local minima of the elevation function, in this case the local minima of the intensity image, are used as seeds.

Often times, certain characteristics of an image such as noise, low contrast variations, and unclear boundary, make it challenging to have a single minima location for each object in the image. For these reasons, the traditional watershed method will produce over segmentation of an image (whereby an image is overly divided into several regions, with a single object divided into multiple regions due to too many local minima in an intensity image [11]). One method that has been proposed to remedy this problem is the hierarchical watershed [13]. The seeded watershed was introduced to curb this difficulty by *a priori* providing a set of seeds for the image. The task of seed finding for each object in an image is a nontrivial one, which can be compared to estimating the number of clusters in a given data [14], and attempts have been made to find accurate seeds for 3D nuclei segmentation. Since manual seed selection techniques are almost impractical in 3D images, the focus of recent research has been on automated methods utilizing some variant of the intensity image. The distance transform function, which computes the Euclidean distance transform of the binarized (foreground assigned true, and background assigned false) image is a very popular technique used for seed finding [7,8]. The transform assigns to every pixel in the foreground its minimum distance to a background pixel. After the distance transform is applied on the binarized image, it is then thresholded (or the local minima is simply used) for seeds. Unfortunately, determining which threshold value to use when attempting to binarize the distance transform can be a tedious trial and error process, and may require extensive user interaction that defeats the purpose of automation and hinders reproducibility of results. The SMMF algorithm [7] uses a preprocessing step where the seeds are computed using an adaptive H-minima transform to suppress spurious local minima obtained from the distance transform and therefore suppress oversegmentation. As the authors point out in [7], this process focuses on minimizing oversegmentation, and may not handle the problem of undersegmentation efficiently. In [15] the authors adopt the extended H-minima transform applied on the input image for seed detection, with a noise level parameter also supplied as input into the transform which defines the level of variation allowed within a regional minima. The noise parameter invariably determines how many seeds are found in the image, which could lead to oversegmentation. The authors therefore adopt a region merging step to suppress this problem. The MINS toolbox [6] introduced a seed finding technique that uses a multiscale blob detection technique based on the Hessian image to identify seeds, and then a scale-space analysis to suppress noise. Then a seeded geodesic segmentation is performed on the image with the computed seeds. This technique is similar in spirit to the proposed method, but differ in theory, since it uses the eigenvalues of the Hessian. The resulting characterization of ‘seed’ regions is less accurate and leads to segmentation results that do not retain original shape of cells. In this paper, we propose a scheme based on the principal curvatures of the image for finding seeds. The technique of using spectral characterization of surfaces for segmentation is not entirely new. In [16], the authors use the eigenvalues of the hessian of a 2D image as the input to the watershed algorithm. Similar approaches are used in the 3D case in [6]. From theoretical standpoint, the use of the eigenvalues of the Hessian to approximate the principal curvatures leads to less accurate segmentation as far as retaining original shape of cells. As we will present in Section 3, the Hessian tensor and the Shape operator are entirely different, except at critical points on the surface. In [17], an improved estimation of the curvature of 3D meshes (2-manifolds embedded in 3D space) was introduced. The authors report improved accuracy of the estimated curvature by showing results of the watershed segmentation applied on the mean curvature. This method does not explicitly estimate the principal curvatures, but algebraic combinations such as the mean. The method introduced in this paper is similar to these curvature methods, but derives from the intuition about the spectrum of the Shape operator. The eigenvalues of the Shape operator characterizes the points on an embedded surface in a manifold. For DAPI stained nuclei, regions of relatively high intensities can be identified as the objects of interest, namely the nuclei. To obtain the centers of these nuclei, we utilize concepts from differential geometry. The 3D image is interpreted as a 3D-manifold embedded in a 4D space. The physical characterization, or shape, of the manifold (interpreted as a morphological landform) is graphically illustrated in the 2D case (as a 3D surface), and will offer an intuitive way of describing the 3D case. An obvious caveat is that for 2D images with high intensity variations in the interior, the absence of smoothness will cause multiple seeds to be detected. This problem is mitigated in the 3D case since such variations do not propagate throughout the 3D volume unless there are obvious boundaries. Another way we obviate such problem is by mollifying the original image (smoothing the image with a Gaussian kernel).

The remainder of the paper is organized as follows: In Section 2 we briefly introduce the mathematical formulation of the watershed transform. In Section 3 we give a brief introduction to this geometric characterization of curves and manifolds, with illustrative examples for a 1D curve and a 3D surface. The stages leading up to the segmentation is also illustrated, first with synthetic data. Section 4 presents the methods and materials used for evaluation. In Section 5 we present and discuss the performance of the method compared with MINS, SMMF and CellSegm. We also highlight the computational load of the method and provide suggested heuristics for applying the method on large images. We conclude in Section 6.

## 2. The Watershed Transform

The watershed transform was originally introduced by Digabel and Lantuejoul [11,18] in 1978. It was first used as a tool for contour detection by Beucher and Lantuejoul [19], and later applied in image segmentation by Beucher and Meyer [12,20]. The method presented in this paper is based on the flooding method by Meyer [21]. The distance function used in defining the watershed transform in this case is the *Topographical distance*. Traditionally the watershed transform is computed on the morphological gradient of the image [11,21]. We present the definition of the watershed transform in the continuous case. Several definitions which address common problems in the discrete case can be found in [11].

### Definition 1. Topographical distance

*Let C*(*D*) *be the space of twice continuously differentiable functions on a connected domain D with only isolated critical points. Suppose that f* ∈ 𝒞(*D*). *Then the topographical distance T_f_* (*p, q*) *between p, q* ∈ *D is defined by*:

$$T_{f}(p,q)=\underset{\gamma }{\text{inf}}\int_{\gamma }\|\nabla f(\gamma (s))\|ds,$$

*where* γ *is any smooth curve in D satisfying* γ(0) = *p*, γ(1) = *q*).

### Definition 2. Catchment Basin

*Suppose f* ∈ *C*(*D*) *has a set of minima* {*m_i_*}_*i*∈*I*_, *for some indexed set I. The catchment basin CB*(*m_i_*) *of a minimum m_i_ is defined as the set of points x* ∈ *D which are topographically closer to m_i_ than to any other m_j_*:

$$CB(m_{i})=\{x|\forall j\neq i:f(m_{i})+T_{f}(x,m_{i})<f(m_{j})+T_{f}(x,m_{j})\}.$$

### Definition 3. Watershed transform

*Given the set of catchment basins of f, the watershed, Wshed*(*f*), *of f is defined as the set of points not belonging to any catchment basin*

$$\text{Wshed}(f)=D\cap {\left(\underset{i\in I}{⋃}CB(m_{i})\right)}^{c},$$

*where* (·)^*c*^ *denotes the compliment of a set*.

Now for such points *p* ∈ *Wshed*(*f*), assign a label *w*. The watershed transform then finds a unique label *i* for each catchment basin *CB*(*m_i_*) such that *i* ≠ *w*. For some label *w* ∉ *I*, the watershed transform of *f* is a mapping λ : *D* → *I* ∪ {*w*} such that

$$\lambda (p)=\{\begin{array}{cc} i, & \text{if }p\in CB(m_{i})\\ w, & \text{if }p\in \text{Wshed}(f) \end{array}.$$

Our method is motivated by the following definition of the *topographical distance from a point to a set*.

### Definition 4. Topographical Distance (from point to a set)

*Let f* ∈ *C*(*D*) *and* 𝒩_*i*_ *be an open subset of D. Then for any p* ∈ *D, we define the topographical distance between p and* 𝒩_*i*_ *as*

$$T_{f}(p,{𝒩}_{i})=\underset{q\in {𝒩}_{i}}{\text{inf}}T_{f}(p,q).$$

From the preceeding definitions, the accuracy of the watershed segmentation is largely dependent on the accuracy of the set of minima of the image. Often times an image may have multiple minima in one catchment basin. Instead of using a single location in each catchment basin, the traditional watershed uses the entire set of minima found. In the next section we focus on finding an accurate set of neighborhoods {𝒩*i*}_*i*∈*I*_, one for each catchment basin using the principal curvatures.

## 3. The Shape of the Manifold

In this section we give a brief overview of the differential geometric properties of an embedded 3-surface that are key to our method. In particular, we introduce the *first* and *second fundamental forms*, the associated *Weingarten* map, and the corresponding matrix. The spectral features, namely the eigenvalues (principal curvatures), are effectively used for detecting cell centroids.

An oriented parametric 3-surface Γ (hypersurface of codimension 1) in ℝ can be defined by the set

$$\Gamma =\{{\left(\gamma_{1}(x,y,z),\gamma_{2}(x,y,z),\gamma_{3}(x,y,z),\gamma_{4}(x,y,z)\right)}^{T}\in {\mathbb{R}}^{4}\}$$

where (*x, y, z*)^*T*^ ∈ ℝ^3^, and γ_1_, γ_2_, γ_3_, γ_4_ ∈ 𝒞^2^(ℝ^3^, ℝ). It is not uncommon to represent Γ as encoded by the parametrization **X**(*u*, υ, *w*), where **X** is a vector-valued 𝒞^2^ function, and (*u*, υ, *w*) some appropriate coordinate system.

### 3.1. The First and Second Fundamental Forms

#### Definition 5

*Consider the surface* Γ ⊂ ℝ^4^, *with parametrization* **X**(*u*, υ, *w*). *The first fundamental form is the quadratic form on the tangent space I_p_* : *T_p_*(Γ) → ℝ, *defined by*

(1) $$I_{p}(w)=\langle w,w\rangle ={\left\|w\right\|}^{2}$$

*where T_p_*(Γ) *is the tangent plane to* Γ *at the point p and* **w** ∈ *T_p_*(Γ).

Considering **w** ∈ *T_p_*(Γ) as being tangent to some parametrized curve on *S*, that is **w** = **X**′(*u*, υ, *w*)(*p*), Equation (1) can be expressed in terms of the basis {**X**_*u*_, **X**_υ_, **X**_*w*_} (associated with the parametrization above, and choosing **w** = λ_1_**X**_*u*_ + λ_2_**X**_υ_ + λ_3_**X**_*w*_) as follows:

(2) $$I_{p}(w)=\lambda_{1}^{2}E+2\lambda_{1}\lambda_{2}F+2\lambda_{1}\lambda_{3}H+\lambda_{2}^{2}G+2\lambda_{2}\lambda_{3}J+\lambda_{3}^{2}K,$$

where *E, F, G, H, J* and *K* are given by:

$$E={\langle X_{u},X_{u}\rangle }_{p},F={\langle X_{u},X_{\upsilon }\rangle }_{p},H={\langle X_{u},X_{w}\rangle }_{p},G={\langle X_{\upsilon },X_{\upsilon }\rangle }_{p},J={\langle X_{\upsilon },X_{w}\rangle }_{p},K={\langle X_{w},X_{w}\rangle }_{p}.$$

In metric tensor form, the *first fundamental form* is represented as the symmetric matrix:

$$I_{p}=(\begin{array}{ccc} E & F & H\\ F & G & J\\ H & J & K \end{array}).$$

#### Definition 6

*The Gauss map is the map*

$$N:\Gamma \to S^{3},$$

*where S*^3^ *is the unit 3-sphere. The Gauss map assigns, to every point the corresponding unit vector on the unit sphere S*^3^. *For any given curve c*(*t*) ∈ Γ, *where c*(0) = **p**, *the differential d***N**_*p*_ : *T_p_*(Γ) → *T_p_*(Γ) *of the Gauss map at* **p** *measures the rate at which the normal vector* **n** *changes direction. This is an endomorphism of the tangent space T_p_*(Γ) [22] *called the Weingarten map*.

#### Definition 7

*The quadratic form II_p_, defined in T_p_*(Γ) *by*

(3) $$II_{p}(w)=-\langle dN_{p}(w),w\rangle$$

*is called the second fundamental form of* Γ *at* **p**.

In terms of the basis {**X**_*u*_, **X**_υ_, **X**_*w*_}, the *second fundamental form* can be expressed as (again writing **w** = μ_1_**X**_*u*_ + μ_2_**X**_υ_ + μ_3_**X**_*w*_)

$$II_{p}(w)=\mu_{1}^{2}L+\mu_{2}^{2}N+\mu_{3}^{2}R+2\mu_{1}\mu_{2}M+2\mu_{1}\mu_{3}O+2\mu_{2}\mu_{3}Q$$

where *L, M, N, O, Q* and *R* are given by

$$L=-\langle n_{u},X_{u}\rangle =\langle n,X_{uu}\rangle$$

$$M=-\langle n_{\upsilon },X_{u}\rangle =\langle n,X_{u\upsilon }\rangle =\langle n,X_{\upsilon u}\rangle =-\langle n_{u},X_{\upsilon }\rangle$$

$$O=-\langle n_{u},X_{w}\rangle =\langle n,X_{uw}\rangle =\langle n,X_{wu}\rangle =-\langle n_{w},X_{u}\rangle$$

$$Q=-\langle n_{\upsilon },X_{w}\rangle =\langle n,X_{\upsilon w}\rangle =\langle n,X_{w\upsilon }\rangle =-\langle n_{w},X_{\upsilon }\rangle$$

$$R=-\langle n_{w},X_{w}\rangle =\langle n,X_{ww}\rangle$$

$$N=-\langle n_{\upsilon },X_{\upsilon }\rangle =\langle n,X_{\upsilon \upsilon }\rangle$$

with the unit normal

$$n=\frac{X_{u}\times X_{\upsilon }\times X_{w}}{\left|X_{u}\times X_{\upsilon }\times X_{w}\right|}.$$

The metric tensor form of *II_p_* is given by the symmetric matrix:

$$II_{p}=(\begin{array}{ccc} L & M & O\\ M & N & Q\\ O & Q & R \end{array}).$$

Since **n**_*u*_, **n**_υ_, **n**_*w*_ ∈ *T_p_*(Γ), it follows that (taking as basis for *T_p_*(Γ), {**X**_*u*_, **X**_υ_, **X**_*w*_})

(4) $$(\begin{array}{c} n_{u}\\ n_{\upsilon }\\ n_{w} \end{array})=A(\begin{array}{c} X_{u}\\ X_{\upsilon }\\ X_{w} \end{array}).$$

where *A* is the matrix known as the *Shape Operator* or *Weingarten operator*. The components of *A* can be obtained from the *first and second fundamental forms*, first by post multiplying (4) by (**X**_*u*_, **X**_υ_, **X**_*w*_)^*T*^ and making *A* the subject [23]

(5) $$A=-II_{p}I_{p}^{-1}.$$

The eigenvalues of *A* are the *principal curvatures*, whose product is the *Gaussian curvature* and average gives the *Mean curvature*.

### 3.2. Curvature of Implicit Surfaces

Our goal is to segment a given 3D intensity image. This image can be interpreted as the level function *t* = *f*(*x, y, z*), where *t* is the intensity value at the point (*x, y, z*). Therefore the image can be thought of as an embedded surface in ℝ^4^, with the parametrization **X** = (*u*, υ, *w, f*(*u*, υ, *w*)) by letting *u* = *x*, υ = *y, w* = *z*.

The first order partial derivatives are the vectors

$$X_{u}=(1,0,0,f_{u}),X_{\upsilon }=(0,1,0,f_{\upsilon }),X_{w}=(0,0,1,f_{w}),$$

and the second order partials are also defined accordingly.

The unit normal is then computed as

(6) $$n=\frac{\left(-f_{u},-f_{\upsilon },-f_{w},1\right)}{\sqrt{1+f_{u}^{2}+f_{\upsilon }^{2}+f_{w}^{2}}}.$$

From the parametrizations of **X**_*u*_, **X**_υ_, **X**_*w*_ and the normal vector Equation (6), it follows that the *shape matrix* is

$$A=\frac{1}{l}(\begin{array}{ccc} f_{uu} & f_{u\upsilon } & f_{uw}\\ f_{\upsilon u} & f_{\upsilon \upsilon } & f_{\upsilon w}\\ f_{wu} & f_{w\upsilon } & f_{ww} \end{array}){\left(\begin{array}{ccc} 1+f_{u}^{2} & f_{u}f_{\upsilon } & f_{u}f_{w}\\ f_{u}f_{\upsilon } & 1+f_{\upsilon }^{2} & f_{\upsilon }f_{w}\\ f_{u}f_{w} & f_{\upsilon }f_{w} & 1+f_{w}^{2} \end{array}\right)}^{-1},$$

where

$$l=\sqrt{1+f_{u}^{2}+f_{\upsilon }^{2}+f_{w}^{2}}.$$

In passing, the *Gaussian curvature K_g_*, is the product of the *principal curvatures* (the eigenvalues of *A*). That is, *K_g_* = det(*A*),

$$K_{g}=\text{det}(A)=\frac{f_{uu}f_{\upsilon \upsilon }f_{ww}-f_{ww}f_{u\upsilon }^{2}-f_{\upsilon \upsilon }f_{uw}^{2}-f_{uu}f_{\upsilon w}^{2}+2f_{u\upsilon }f_{uw}f_{\upsilon w}}{{\left(1+f_{u}^{2}+f_{\upsilon }^{2}+f_{w}^{2}\right)}^{5/2}}.$$

Similarly, the *Mean curvature* is given by the divergence of the unit normal:

$$K_{m}=-\nabla N_{p}=\frac{1}{l}(f_{uu}(1+f_{\upsilon }^{2}+f_{w}^{2})+f_{\upsilon \upsilon }(1+f_{u}^{2}+f_{w}^{2})+f_{ww}(1+f_{u}^{2}+f_{\upsilon }^{2})-2(f_{u}f_{\upsilon }f_{u\upsilon }+f_{u}f_{w}f_{uw}+f_{\upsilon }f_{w}f_{\upsilon w})).$$

**Figure 1:**
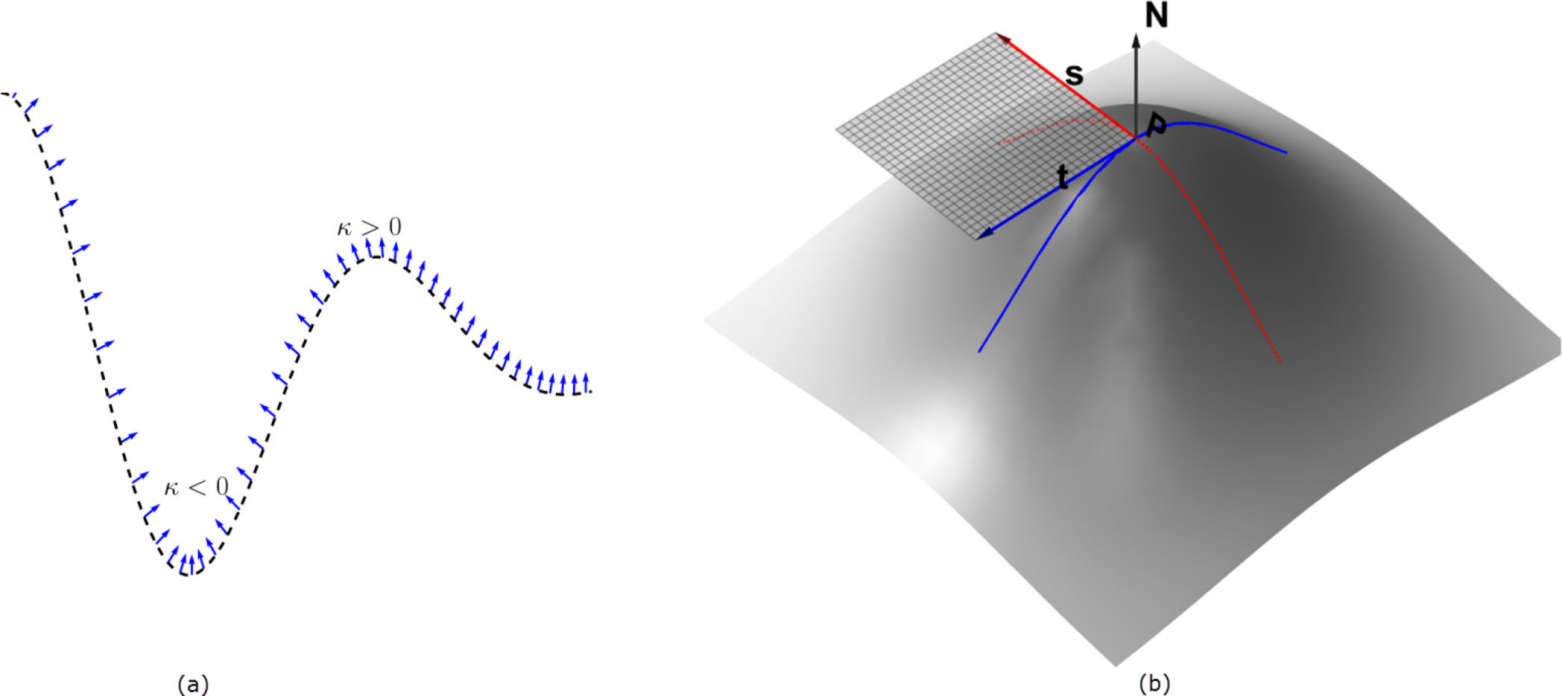
In panel (**a**), a parametric curve in ℝ is shown with the normals at sampled points. The region where the normals are converging has negative curvature, while the opposite is true for where the normals diverge. In panel (**b**), a parametric surface is depicted, with the two principal directions **s**(= *X_u_*) and **t**(= *X*_υ_) at the point **P**. A portion of the tangent plane to the surface at the point P is also shown in transparent grid, as determined by **s** and **t**, and the corresponding normal $N=\frac{s\times t}{\left|s\times t\right|}$ is the indicated up arrow. The principal directions correspond with the maximum and minimum curvature directions on the surface. All of these images are better visualized in color.

**Figure 2:**
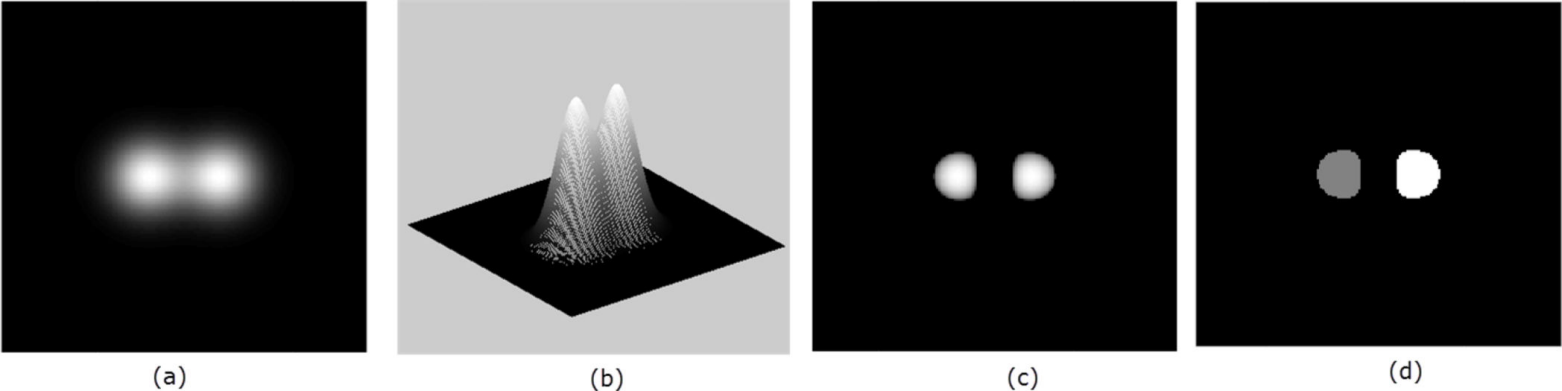
Panel (**a**) shows a 2D image, while panel (**b**) shows the same image as an embedded surface. In panel (**c**), the detected seeds are shown. Panel (**d**) shows the labels for the two seeds in (**c**).

The signs of the principal curvatures, *k*_1_, *k*_2_, *k*_3_, are used to characterize the points on the surface. At the points where *k*_1_, *k*_2_, *k*_3_ ≥ 0, the surface is locally convex and it bends outward in the direction of the chosen normal **n** (Figure 1). This follows from the fact that the principal curvatures are the stretching factors on the surface in the direction of the respective principal direction. The intuition can easily be visualized for 2D image (visualized as 3D surface), in which case there are only two principal curvatures *k*_1_, *k*_2_. In the characterization of the surface in Figure 2b, the neighborhood of the peaks form a ‘dome’ shape. Thus by choosing an upward pointing normal **n**, all parametric curves through a point on the ‘dome’ will have positive curvature and hence the shape is characterized by by *k*_1_, *k*_2_ ≥ 0. So for an image with higher intensities corresponding to object locations, the points with identically positive principal curvatures identify with approximate object location. We therefore obtain the seeds *Ct* of the ‘objects’ in Figure 2a by taking the product

$$Ct=k_{1}^{+}k_{2}^{+},\text{ where }k_{i}^{+}=\text{max }\{k_{i},0\},$$

which is then clustered for distinct centers as shown in Figure 2b. In the case of 3D images *f*(*x, y, z*) = *t*, we compute *Ct* as defined but this time with the three principal curvatures *k*_1_, *k*_2_, *k*_3_:

$$Ct=k_{1}^{+}k_{2}^{+}k_{3}^{+}\text{ where }k_{i}^{+}=\text{max }\{k_{i},0\}.$$

**Figure 3:**
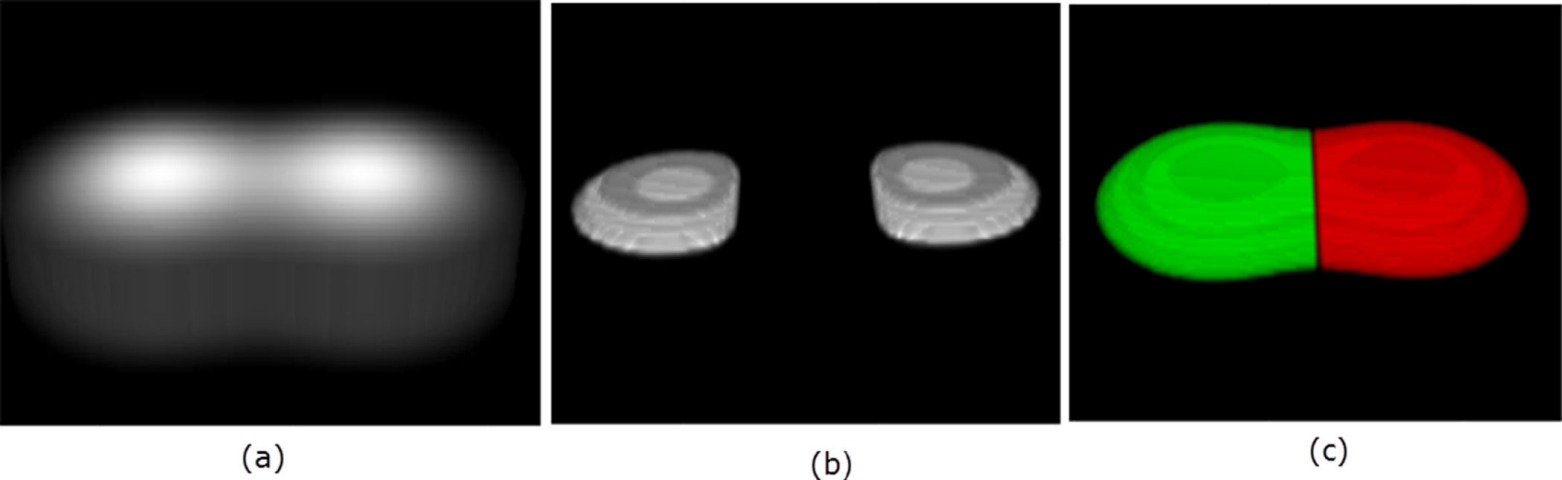
3D rendering of two objects in (**a**). The located seeds, $Ct=k_{1}^{+}k_{2}^{+}k_{3}^{+}$, are visualized in 3D in panel (**b**). The final segmentation is shown in panel (**c**).

The same analogy holds for the 3D embedded manifold. That is, the points of positive principal curvatures correspond with high intensities, and therefore identify where objects are located. An example is illustrated in Figure 3 with two touching Gaussians defined on a grid of size 200 × 200 × 15, with the resulting clustering and segmentation shown.

## 4. Materials and Methods

### 4.1. Dataset

Two sets of data were used to evaluate the proposed method: Experimental (simulated) and 3D images of Drosophilia larval brains. The 3D images of Drosophilia larval brains were obtained by scanning in a confocal microscopy. We used the G-trace clonal method (Evans et al., 2009) to mark distinct cell lineages expressing Green Fluorescent Protein (GFP) and Red Fluorescent Protein (RFP). In this genetics system, late-born cells express RFP, while intermediate cells express a mix of RFP and GFP, and early-born cells express GFP. The images were collected with a resolution of 1024 × 1024 pixels and the confocal stacks contained roughly 60 slices of 0.45 µm of thickness each for WILD TYPE larval brains, and 55 slices of 0.45 µm of thickness each for MUTANT TYPE larval brains. The staining scheme employed generates a nuclei-stained volume, and therefore very well-suited for the method. In all such images, we took the sum of GFP and RFP to be the input of the segmentation algorithm.

The simulated dataset were generated by Svoboda et al. [24], to simulate the HL-60 cell line using the CytoPacq toolbox. Initially, the outline or shape of the cell nuclei are taken to be spheres or ellipsoids, and then a PDE-based method is used to deform the shape to become irregular and less convex, by deforming the boundary (as a deformable surface). Based on expert knowledge texture is incorporated by applying several Perlin noise functions on the deformed body, to give its internal structure realistic semblance to the HL-60 cell line. Further details can be found in [24].

### 4.2. Evaluation Methodology

The simulated data also comes with ground truth labeled objects, so some quality assessment index can be used to evaluate the method and compare with other methods. We compare with the MINS module [6] which consist of an initial nuclei detection, followed by watershed segmentation using the initial nuclei as seed. The toolbox performs seed detection using multiscale blob detection. CellSegm is aMATLAB toolbox which assembles hybrid image segmentation techniques, with adaptive thresholding and watershed segmentation being notable components. The toolbox also performs a post processing step via splitting of undersegmented nuclei based on user-supplied parameter related to the estimated average size of nuclei. We also compare with the SMMF algorithm, which consist of seed detection using the H-minima transform, followed by the seeded watershed segmentation.

We use some standard metrics that give quantitative measure of the accuracy of the segmentation methods, namely, the *Rand Index* [25] and the *Jaccard Index* [26]. The *Rand Index* measures similarity between ground truth labels *L* and segmentation results *S*, using the ratio of matching labels in *L* and *S* to the total volume. It is usually converted to percentage, and is closer to 100% for very accurate segmentation. The Jaccard Index is defined by the ratio of the size of the intersection between labels of *L* and *S* to the size of their union. This measure is typically between 0 and 1, with 1 being perfect segmentation. In addition to these metrics, the results were checked for occurrence of oversegmentation and undersegmentation. If ground truth object *A* has been split into parts B and C in the segmented image, then we say *A* is oversegmented if the volume of *B* and *C* are both less than 80%. That is, neither *B* nor *C* occupies 80% of *A*. We also define the number of undersegmented nuclei as the number, *N*, of nuclei in the ground truth image that were merged during segmentation into a single nucleus. Moreover, there were scenarios where objects were missing, so each method was checked for missing objects. This was counted simply as the number of the nuclei that were missing. The values for each index in Table 1 were computed as the average across all (different) volumes in the dataset. For each volume, the values of the *Oversegmented, Undersegmented* and *Missing* nuclei were converted as a fraction of the number of nuclei in the volume. Then the average was taken over all volumes.

Due to the non-availability of reliably segmented ground truth 3D electron microscopy images, we are only able to present qualitative results on such data. The image data thus used is a segment of Dapi stained nuclei of Drosophilia larval brains scanned in confocal microscopy.

### 4.3. Data Pre-processing

**Figure 4:**
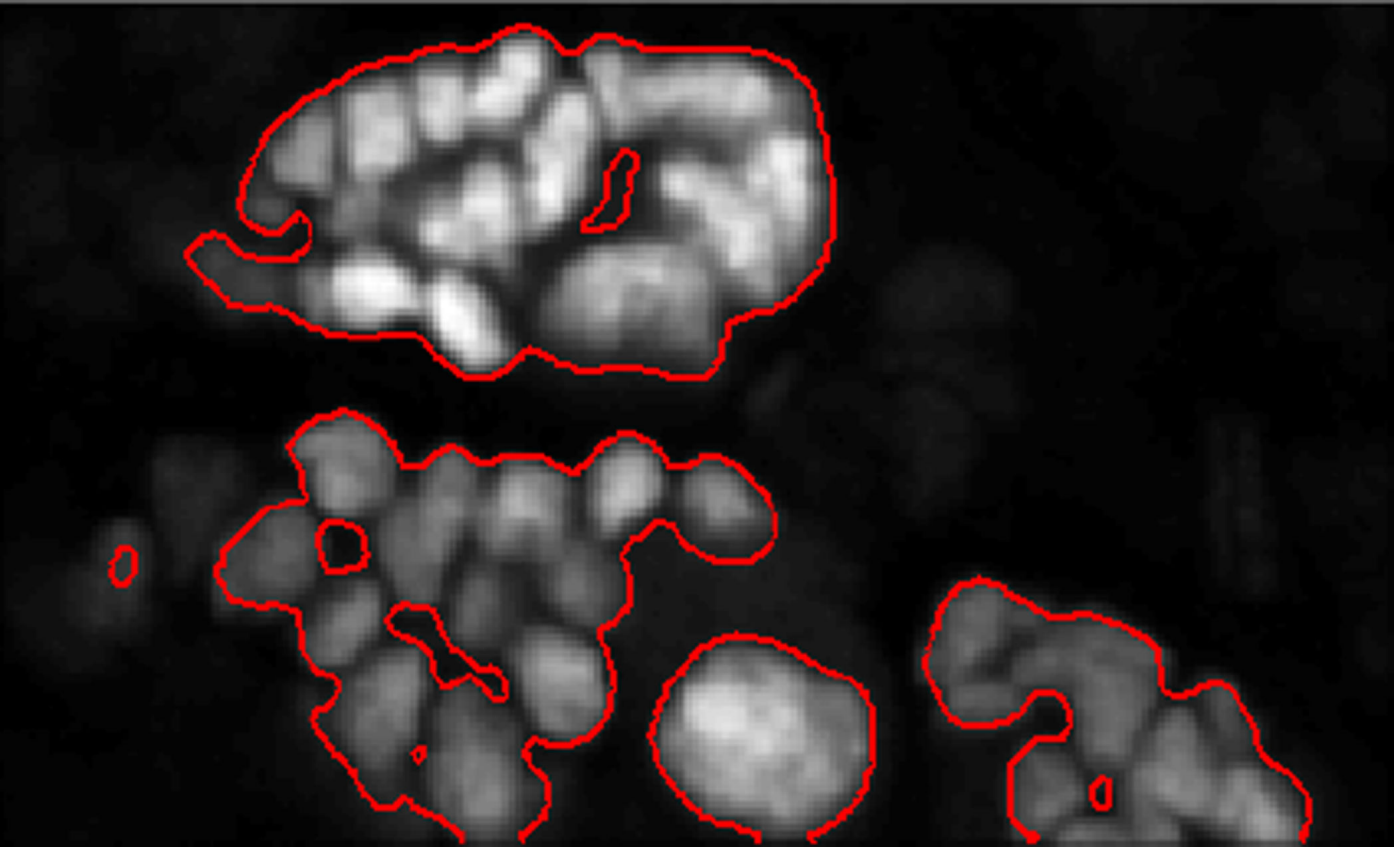
A superposition of the bounding contours on the original image.

In the implementation of the method, an important step to computing the seeds is to restrict the region of interest to the cell nuclei. More importantly, since this approach uses the watershed algorithm, it is necessary to obtain a foreground mask of the objects in the image. The watershed method is exhaustive in that the entire domain is partitioned. To restrict the partition to objects of interest, we adopt the ‘Chan-Vese’ algorithm [3], which uses the theory of evolving curves to encapsulate objects found in images. Granted its robustness, the method can only group objects together when there are no clear boundaries between them. This method enables us to automatically detect cell colonies in the image. For a detailed description of the ‘Chan-Vese’ method, we refer the interested reader to [3]. In Figure 4, the bounding contours resulting from the ‘Chan-Vese’ are shown superimposed on the original image.

### 4.4. Seed Detection and Watershed

After restricting the domain of partition to the cell colonies, we then compute the cell centroids using the curvature formulas in the previous section. An important underlying assumption for the curvature formulas given in Section 3 is that *f*(*x, y, z*) = *t* is smooth enough (at least up to second order). The problem with discrete images is that noise can become a nuisance, which undermines regularity. The image is therefore convolved with some kernel *g* (at least twice continuously differentiable), such as a Gaussian. The importance of this step is two-fold: First, it allows us to compute the higher order derivatives by shifting the derivatives onto *g*, since *f* ∈ *L*^1^, and *g* is continuously differentiable with compact support. Secondly, since the convolution with a Gaussian can be seen as an averaging operation, noise is sufficiently smoothed out. Beyond this step, all computations are carried out using *f* * *g*, the convolution of *f* and *g*.

After computing the eigenvalues there is a potential challenge of spurious seeds in the product $Ct=k_{1}^{+}k_{2}^{+}k_{3}^{+}$. As a precaution, we apply a morphological erosion on *Ct* with a 3 × 3 × 3 structuring element *h* defined below:

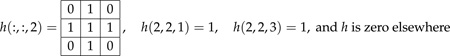



**Figure 5:**
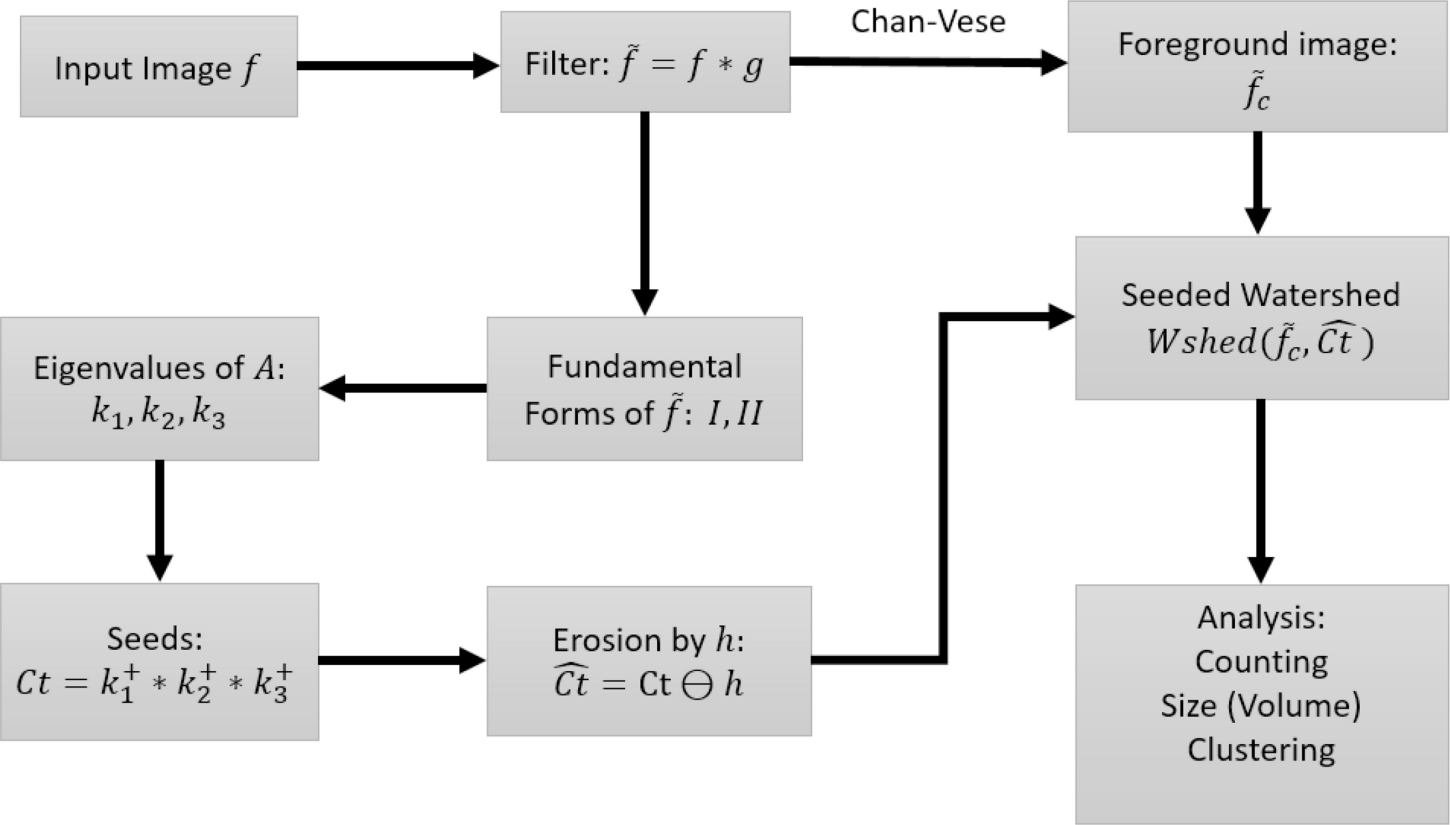
Flow of the processing steps in the proposed method.

**Figure 6:**
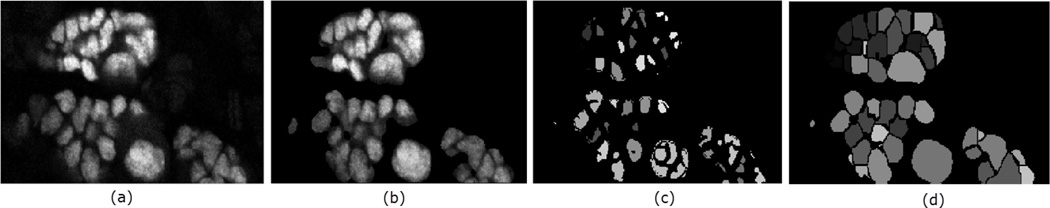
Illustration of the key steps in the proposed segmentation method. Panel (**a**) is a slice of the original volume to be segmented; (**b**) is the resulting foreground from the chan-vese method; In panel (**c**) the obtained seeds are shown; while the resulting segmentation is shown in (**d**).

The erosion not only eliminates spurious seeds, but is also able to break falsely connected centroids (those with thin necks). The size of the structuring element, *h*, has to be adjusted to suit the properties of the image. For heavily clumped nuclei, a larger *h* is required. (MATLAB Image Processing Toolbox has built-in functions for bigger structuring elements). After obtaining the seeds in *Ct*, the watershed algorithm is applied to *f* * *g* (the blurred image), with *Ct* as the set of seeds for the foreground objects. In our implementation we do not explicitly provide seeds for the background, as it is unnecessary. For any nuclei that border the background, the watershed algorithm will grow from the seed of the nuclei, and stop at the boundaries of neighboring nuclei. Towards the direction of the background however, it will grow into the background. At the end of the watershed algorithm, we again impose the extracted foreground image obtained by the active contour method of [3], which restricts the segmented image to the colonies obtained from the preprocessing stage. The watershed implementation is based on [21], and the input image is converted to the norm of the gradient, which becomes the marking function, with object boundaries having higher gradient values. Since the seeds set is now a collection of connected neighboring pixels instead of single pixels, the accuracy of the watershed lines is also greatly improved. More importantly, the shape or outline of cell nuclei is inherited by the computed seeds using the spectral properties of the shape matrix, namely the eigenvalues. To this point, the process can be summarized in Figure 5, with selected nodes in the process shown in Figure 6.

## 5. Results and Discussion

We collected a total of 30 volumes of the simulated HL-60 cell line [24], with low Signal-to-Noise Ratio and 75% probability of clustering (relatively more clustered nuclei). Each image volume has dimension 807 × 565 × 129, containing 20 nuclei. The parameters for each algorithm were optimized using a fixed set of 3 volumes to select optimal parameter combinations for each method over a range of the values as guided by the documentation of each respective algorithm.

### 5.1. Evaluation on Sample Dataset

**Figure 7:**
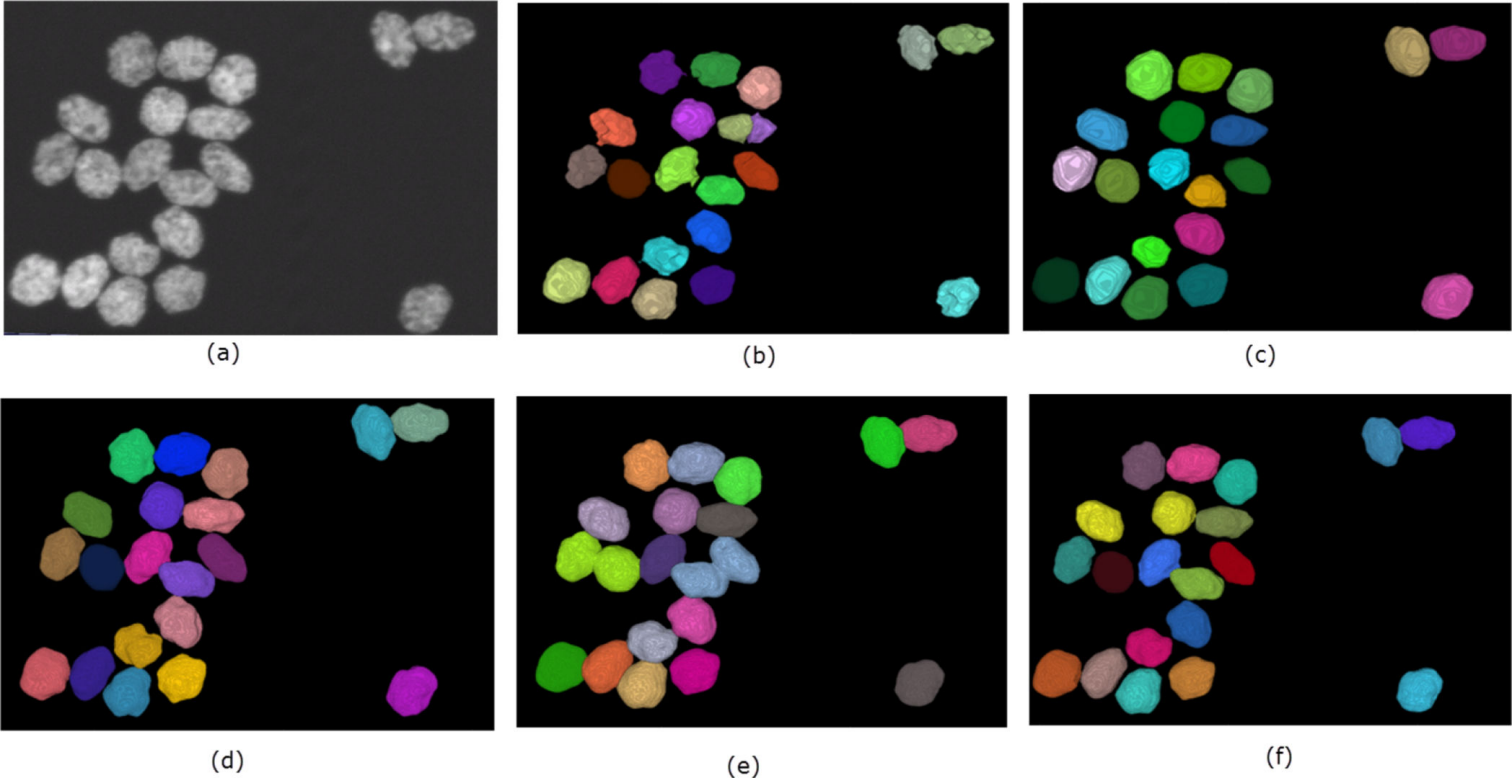
Qualitative performance of the 4 methods on a selected volume of simulated HL-60 cell line. Panel (**a**) shows the original volume. The nuclei are randomly color-coded to distinguish one from another as generated by each method: (**b**) CellSegm, (**c**) MINS, (**d**) ground truth segmentation, (**e**) SMMF, (**f**) Proposed.

A summary of the performance of the methods on the simulated dataset is shown in Table 1. Overall, SMMF and the proposed method performs better than MINS and CellSegm, achieving *Jaccard Index* scores of 0.804 and 0.812 respectively. They also achieve higher *Rand Index* scores (96.57% and 97.05% respectively) as well. While the CellSegm toolbox and MINS produce oversegmentation of the objects, on average they achieve better performance in terms of undersegmentation of objects. The SMMF and the proposed method are more conservative, and this leads to undersegmentation on this dataset. While the indexes used are good indicators for the performance of clustering and segmentation algorithms, the result may be misleading since the synthetic dataset is generated using well-controlled parameters, and may loose natural semblance to real biological data (Figure 7 shows the segmentation produced by the methods on a selected volume from the dataset).

**Figure 8:**
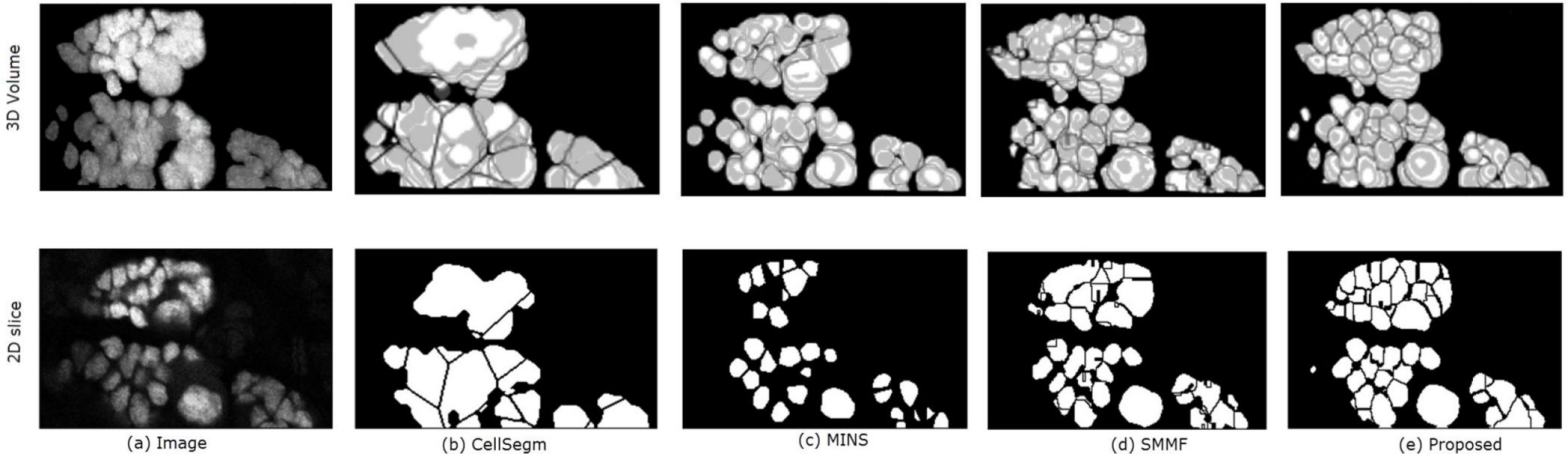
3D Qualitative comparison of the 4 methods on a sample real data. The top and bottom rows show the 3D volume and a selected 2D slice respectively. Panel (**a**) shows the original data. The remaining panels are the segmentation results generated by: (**b**) CellSegm, (**c**) MINS, (**d**) SMMF, (**e**) Proposed method. While the quality indexes suggest good performance on the simulated dataset, the performance on heavily clamped nuclei is low for the CellSegm and SMMF methods. The results from MINS is unable to match the shape of individual cells compared to the proposed method. The first row shows the 3D rendered volume, while the bottom row shows a fixed slice of the volume.

In Figure 8 we show the segmentation results of the four methods using a segment of real data from a Dapi-stained nuclei image. Unlike the simulated images, the nuclei in Dapi-stained image are clamped together not only in the *xy*-directions, but also in the *z*-direction. While CellSegm performs well on objects connected along the *x* − *y* directions, its performance is low on clumped objects, especially where objects neighbor one another in random orientations rather than planar as seen Figure 8b. The MINS performs well in terms of separating the nuclei but unable to suppress multiple seeds for single objects which may lead to small object sizes due to oversegmentation (Figure 8c). Though the SMMF method shows good performance in the 3D visualized segmentation (Figure 8d, row 1), examining the segmentation in 2D slice as shown in the second row of Figure 8d reveals that the problem of undersegmentation is pronounced, where the distinct nuclei are lumped together into one. The proposed algorithm does relatively better by preserving the boundaries of the nuclei in both 2D and 3D, as can been in both rows of Figure 8e.

**Figure 9:**
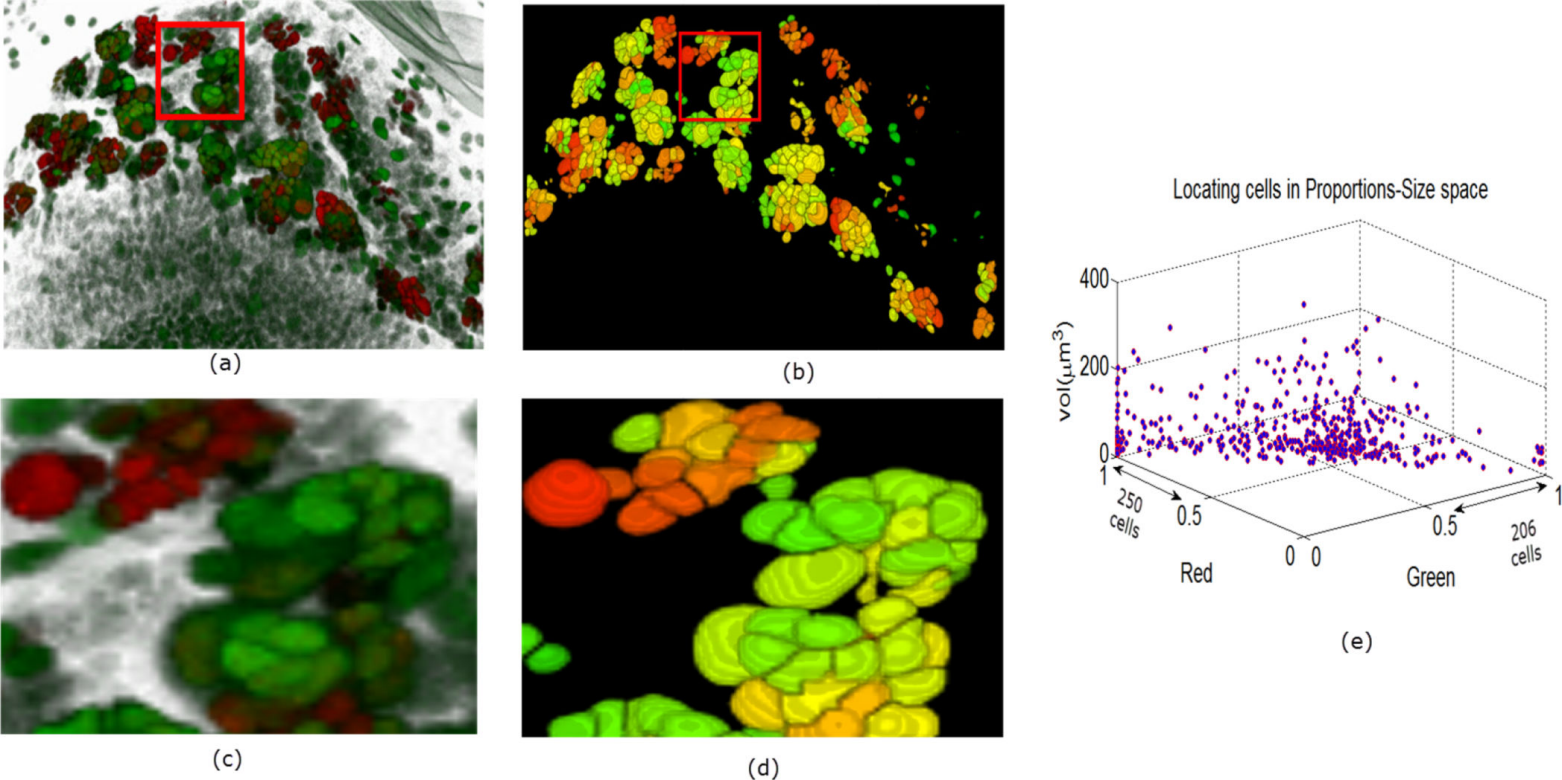
Panel (**a**) shows a 3D view of a WILD TYPE laval brain nuclei. In panel (**b**), the 3D segmentation is rendered in ImageJ. In panels (**c**) and (**d**) enlarged portions are shown from the original and reconstructed images. Panel (**e**) shows the quantitative characteristics of the nuclei in the volume.

**Figure 10:**
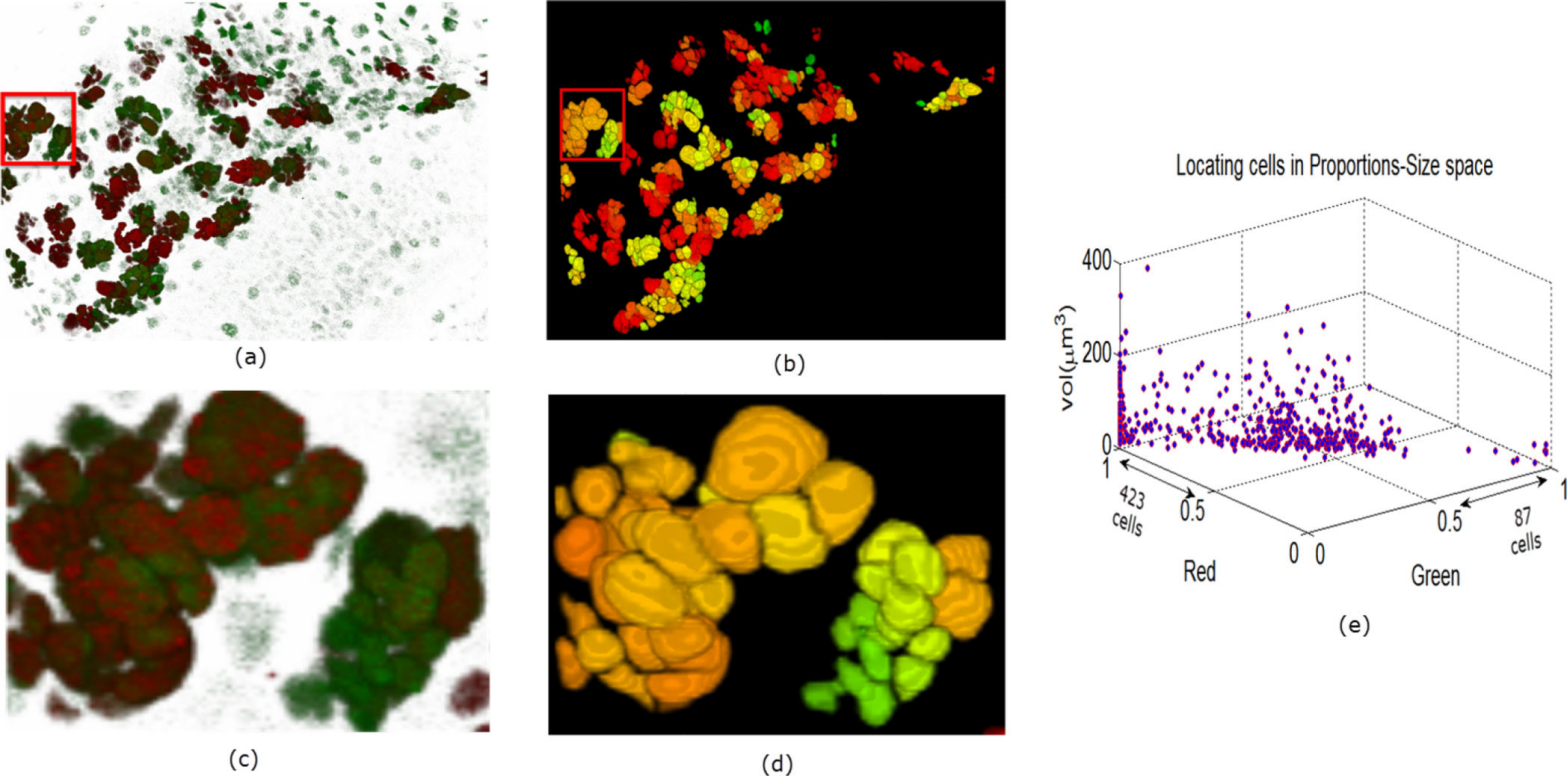
Panel (**a**) is a depiction of a MUTANT TYPE larval brain nuclei in 3D volume in ZEN. In panel (**b**), the 3D segmentation is visualized in ImageJ. In panels (**c**) and (**d**) enlarged regions are shown from the original and reconstructed images. Panel (**e**) similarly shows the quantitative description of the cells.

**Figure 11:**
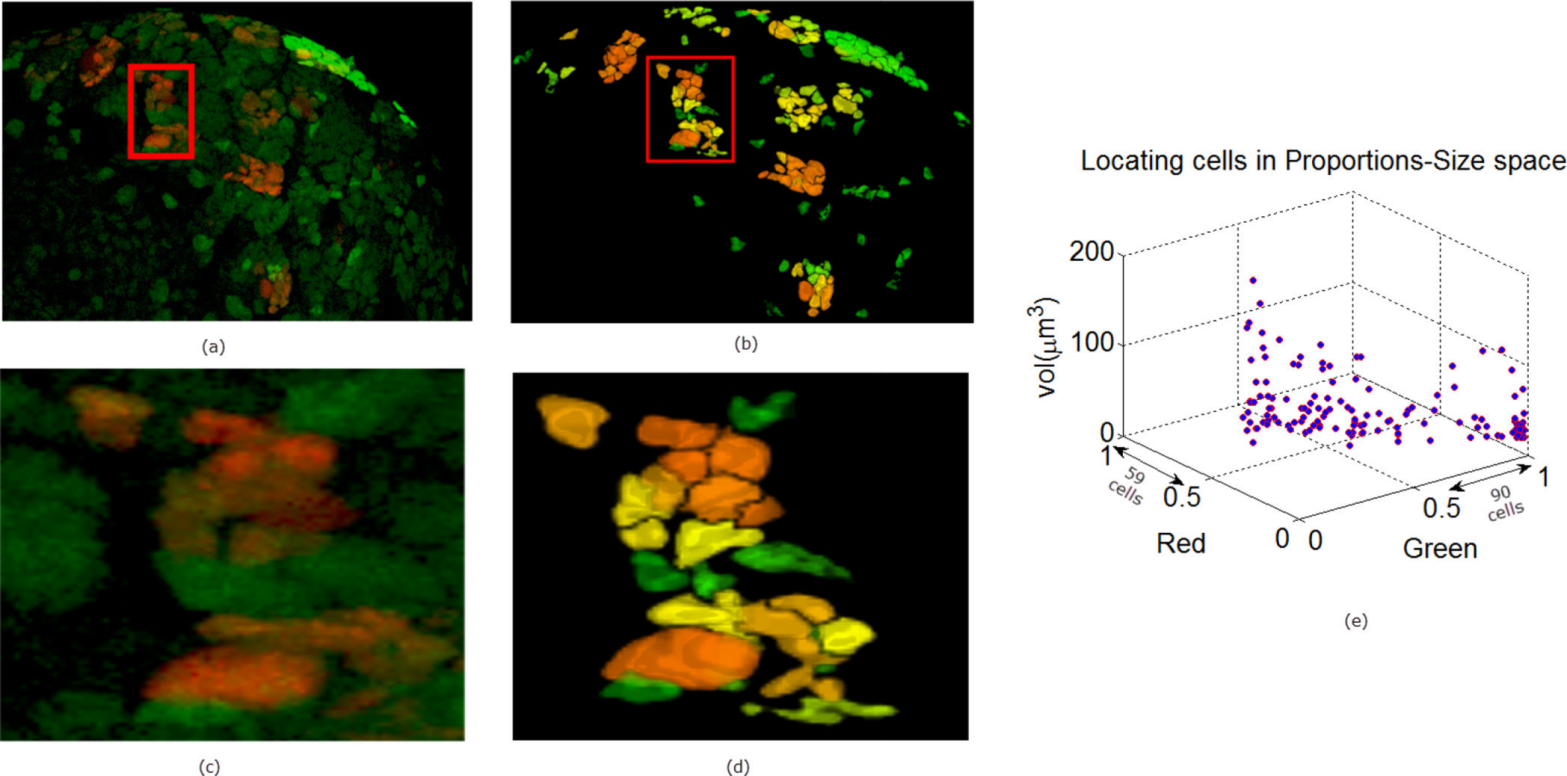
Panel (**a**) is a depiction of another MUTANT TYPE 2 larval brain nuclei in 3D volume in ZEN. In panel (**b**) the 3D reconstruction is shown. The noisy background (GFP) is separated from the true signals using the ACWE [3]. In panels (**c**) and (**d**) enlarged regions are shown from the original and reconstructed images. The clustering of the nuclei in panel (**e**) is based on the segmentation in panel (**a**).

In Figures 9–11 we show some reconstructions of 3D images described in Section 4.1 using the proposed method. To assess the results, the reconstructions are color-coded using the ratio of GFP and RFP. To do this, for each nucleus we take the sum of the RFP and GFP pixel intensities as indicative of the respective proportions of RFP and GFP in the nucleus. For instance, a nucleus that has the ratio of RFP to GFP content close to 1 will be colored yellow (same content of red and green). We also compute the size of each nucleus and create a scatter plot using the computed ratio and the volume in the RFP-GFP-Size space. In Figure 9e, the scatter plot of WILD TYPE larval brains indicate that there is a well-balanced composition of the RFP and GFP in the nuclei (250 nuclei contain more RFP signals than GFP, and the reverse is true for 206 nuclei). The scatter plot of the MUTANT TYPE larval brain in Figure 10e, shows more nuclei with higher RFP content (423 nuclei with higher RFP signal than GFP as opposed to 87 nuclei with more GFP content). In contrast to that of MUTANT TYPE (Figure 10e), the scatter plot of the larval brain clones (Figure 11e) shows a larger number of nuclei with higher GFP content (90 nuclei out of 149).

### 5.2. Computational Issues and Discussions

In terms of computational performance, the proposed method, on average, runs slower than CellSegm and SMMF, while having similar computational complexity as the MINS. The techniques employed in the CellSegm and SMMF lead to cheaper computational cost, roughly half of the runtime of MINS and the proposed method. Both the MINS and the proposed algorithm compute the eigenvalues of a 3 × 3 matrix, which constitutes the largest computational component of these two methods. This will present computational challenges for very large images. On a 3.6 GHz Intel i7, a 1024 × 1024 × 43 image takes on average 1 min to run using the proposed method, while MINS takes on average 50 s to run. Both CellSegm and SMMF runs approximately 30 s or less on same image.

In the proposed method, the eigenvalues of the matrix *A* are computed using MATLAB’s symbolic computing toolbox. The expressions are given in closed form, and thus we can compute *Ct* in one sweep, although that would require a reasonable amount of memory for very large images. For very large images, it is advisable to divide it into blocks of volumes to decrease the memory requirement during computation of the eigenvalues.

Since the *weigarten map* is a self-adjoint operator, and thus has real eigenvalues, we use the real parts of the computed eigenvalues (as numerical computation could add complex component to the eigenvalues).

In our experiments, the size of the smoothing kernel was dependent on the noise level in the image data. For very noisy image data, a significant smoothing has to be done to render the curvature values reliable.

We also emphasize the significance of the erosion by the structuring element *h*. For larger size of *h* a significant amount of seeds will be suppressed. The particular design of structuring elements for different images is not discussed in this study, but useful discussions would be found in [27]. In our experiments, we also found that the morphological erosion by a bigger *h* has the advantage of disconnecting thinly connected seeds, thereby suppressing the problem of undersegmentation. Although we did not observe this phenomenon in our experiments, increasing the size of *h* may potentially cause previously connected seeds to split, leading to oversegmentation. One way to avoid this is to increase the size of the convolution kernel, *g*, before computing the seeds.

## 6. Conclusions

We have presented a seeded watershed that uses the principal curvatures to find seeds for 3D objects. Unlike previous spectral-like segmentation methods, we directly compute the eigenvalues of the Shape operator which intuitively characterize the embedded surface. While there is a close relationship between the Hessian and the Shape matrix of a surface at a given point, that relationship is unique for each point in the domain, and hence the eigenvalues of the Hessian does not accurately represent the principal curvatures of the surface. Using the eigenvalues of the Shape operator instead of the Hessian produces seeds that mimics the overall shape of the cells, which results in segmented cells retaining their original shapes.

The method is automatic, and requires little user interaction for moderately good data. The required input of the algorithm is the structuring element. The performance of the method when compared with other popular packages demonstrates very promising results.

## Figures and Tables

**Table 1:** A comparison of the quality of segmentation on 30 simulated HL-60 cell line images. The values in the quality index are average values over the 30 datasets. In terms of RI and JI, SMMF and the proposed method demonstrate reasonable performance values compared to MINS and CellSegm.

Algorithm	RI	JI	Oversegmented	Undersegmented	Missing
CellSegm	92.24%	0.659	0.005	0.00	0.00
MINS	95.86%	0.713	0.003	0.00	0.025
SMMF	96.57%	0.804	0.00	0.04	0.00
Proposed	97.05%	0.812	0.00	0.015	0.00
